# Recognizing the Frequency of Exposure to Cyberbullying in Children: The Results of the National HBSC Study in Serbia

**DOI:** 10.3390/children11020172

**Published:** 2024-01-29

**Authors:** Milica Kangrga, Dejan Nikolic, Milena Santric-Milicevic, Ljiljana Rakic, Tatjana Knezevic, Goran Djuricic, Jasna Stojkovic, Natasa Radosavljevic, Sladjana Mihajlovic, Biljana Medjo, Milan Lackovic

**Affiliations:** 1 Faculty of Medicine, University of Belgrade, 11000 Belgrade, Serbia; jefticmilica@gmail.com (M.K.); dejan.nikolic@udk.bg.ac.rs (D.N.); tatjana.knezevic@med.bg.ac.rs (T.K.); goran.djuricic@med.bg.ac.rs (G.D.); jasna.stojkovic@med.bg.ac.rs (J.S.);; 2 Department of Physical Medicine and Rehabilitation, University Children’s Hospital, 11000 Belgrade, Serbia; 3 Laboratory for Strengthening Capacity and Performance of Health System and Workforce for Health Equity, Institute of Social Medicine, Faculty of Medicine, University of Belgrade, 11000 Belgrade, Serbia; 4 Clinic for Hematology, University Clinical Center of Serbia, 11000 Belgrade, Serbia; lj.rakic68@gmail.com; 5 Department of Radiology, University Children’s Hospital, 11000 Belgrade, Serbia; 6 Department of Biomedical Sciences, State University of Novi Pazar, 36300 Novi Pazar, Serbia; nradosavljevic@moh.gov.sa; 7 Department of Obstetrics and Gynecology, University Hospital “Dragisa Misovic”, Heroja Milana Tepica 1, 11000 Belgrade, Serbia; milan.lackovic@dragisamisovic.bg.ac.rs; 8 Department of Pediatrics and Neonatal Intensive Care, University Children’s Hospital, 11000 Belgrade, Serbia

**Keywords:** cyberbullying, children, health characteristics, risky behavior

## Abstract

Nowadays, children are able to enrich their reality via the Internet. Unfortunately, this may increase their risk of becoming victims of cyberbullying. We analyzed the health characteristics and risk behavior of two cohorts of children in Serbia; those who reported being exposed to cyberbullying and those who did not. The statistical differences and logistic regression models were applied to the data on 3267 students collected from 64 schools participating in the 2017 Serbian Study on health behavior in school-age children (HBSC). Children exposed to cyberbullying reported having the following health problems on a daily basis: headache (18.5%), back pain (19.5%), depression (21.6%), irritability or bad mood (17.7%), nervousness (16.0%), sleep problems (16.1%), and dizziness (21.2%). As for the different types of risk behavior, cigarette smoking ranging from six to nine days ever was the most prevalent (26.9%). It was followed closely by getting drunk more than 10 times ever (24.1%). Compared to non-victims, victims were found to be at a higher risk of perceived back pain (OR = 2.27), depression (OR = 1.43), irritability or bad mood (OR = 2.07), nervousness (OR = 2.23), and dizziness (OR = 2.43) as well as being injured once or twice (OR = 1.98) or three or more times (OR = 4.09). Victims were associated with further risk factors: having smoked more than five cigarettes ever in life (OR = 1.73) and having gotten drunk two to three times (OR = 1.71) or four or more times (OR = 1.65). As the number of school-age children using social media continues to rise, we must prioritize educating them about self-help and community resources for addressing related health issues with greater speed and intensity. The findings from Serbia suggest that while children may be aware of their health issues, they may be unaware of their link to cyberbullying, which could hinder their ability to address these issues promptly. The respondents’ attention to the health implications of cyberbullying could be increased by reformulating the survey questions used in the HBSC study.

## 1. Introduction

Bullying in children and adolescents is considered as a repeated exposure to negative actions by peer perpetrators, who want to show power over other children and harm the victims [1]. A direct form of bullying typically involves physical attacks on the victim [2], while an indirect form is associated with social isolation [2]. The term bullying is usually defined as “being an aggressive, intentional act or behaviour that is carried out by a group or an individual repeatedly and over time against a victim who can not easily defend him or herself” [2]. In contrast to traditional face-to-face bullying, cyberbullying occurs through some type of media [3], such as social media, messaging platforms, gaming platforms, and mobile phones [4], but these forms are not mutually exclusive. The fact that children can become exposed to cyberbullying more often with an increased use of the Internet and smartphones [3] was the motive of this study to use the representative sample of Serbian schoolchildren to explain the concept of cyberbullying and its definition, frequency, and adverse health events as well as risky behavior in order to facilitate its recognition and counteraction.

The term “cyberbullying” cannot be described with one precise definition; nevertheless, it is defined as “an aggressive intentional act carried out by a group or individual, using electronic forms of contact, repeatedly and over time against a victim who cannot easily defend him or herself” [5,6,7].

An estimated prevalence of cyberbullying depends on the definitions, measurements, and samples that are presented in different studies. Previously it was stated that the peak of cyberbullying is typically in early adolescence [8,9]. The perpetual increase in communication over the Internet has had profound effects on social interactions and relationships. Around one third of individuals who use the Internet are children up to 18 years of age [10]. Some young people have become perpetrators and/or victims of cyberbullying [10]. Cyberbullying victimization rates worldwide are estimated to be between 9% and 40% [11].

In the study by Popović-Ćitić et al., the conducted research in Serbia implied that approximately 10% of the participants in the research were involved in cyberbullying and 20% of the respondents were victims of online bullying [12]. The most frequent ways of cyberbullying were denigration and harassment. The results pointed that the boys, both as the bullies and the victims, were involved in bullying more than the girls. They stated that over 25% of the respondent boys were the victims of cyberbullying [12].

Cyberbullying involves recurring behavior that aims to scare, anger, or shame the intended victim [13], for example, by spreading rumors about someone or posting someone’s embarrassing photos or videos. Other forms of cyberbullying include sending hurtful, abusive, or threatening messages [13]. The cyberbullying often lowers the victims’ self-esteem, increases their depression, and poses significant life challenges [14]. The victims usually experience numerous psychosocial problems, for instance, they can be worried, threatened, frustrated, depressed, or angry about the cyberbullying and engage into substance abuse as a coping mechanism [15]. The victims report more frequent health, emotional, and school-related problems together with poorer relationships with classmates in comparation with those who have not been exposed to bullying [16,17]. Furthermore, cybervictims are more likely to be adolescents who have a poor relationship with their parents [18]. The school-related problems can be reflected in lower grades, missing or disliking school, peer relationship problems, suspension, etc. [15].

Certain personality characteristics such as extraversion and emotional instability were found to be associated with bullying behavior [9,19]. Adolescent cybervictims tend to report psychosomatic problems such as headaches, sleep problems, and recurring abdominal pain [20]. In line with this, previous reports have stated that children that have been cyberbullied present with higher rates of depression and anxiety, emotional distress, along with substance abuse compared to their nonbullied peers [21]. They often feel unsafe and not cared for by the school and wider society [11,20,22].

Being the target of bullying has been linked to depression more than all other outcomes and consequences, besides its strong relation with anxiety [9,23]. Non-suicidal self-harm has also been linked to cybervictimization [24]. Cyberbullying involves recurring victimization over time, which allows for long-term consequences to develop. In the most extreme cases, young victims end up deeply depressed and suicidal.

Cyberbullicide is a new term coined for a subclass of suicides, which are related to cyberbullying [25]. Suicidal ideation and suicide attempts are known effects of bullying [25,26]. Cyberbullies, on the other side, often exhibit criminal behavior and habits, excessively consuming stimulants such as cigarettes and alcohol, engaging in substance abuse, and experiencing social withdrawal [17,25].

Moreover, in previous reports, it was stated that increased levels of anxiety, depression, self-harm, and suicidal behavior are noted in cyberbullying victims compared to those in traditional bullying victims in children and adolescents [3]. Additionally, in the study of Fabris et al., it was noted that there could be a higher impact of cyberbullying than traditional bullying on the well-being of adolescents; thus, exploring potential risk factors could lead to a better planning of prevention and intervention programs [8].

Furthermore, in the study of Marciano et al., the results of the meta-analysis found that there is evidence that bullies who applied the traditional way of bullying have become cybervictims, while the cyberbullies during the time have become victims of the traditional way of bullying. These results point out that there is a vicious bully–victim circle [4].

In this study, we hypothesized that children exposed to cyberbullying once or multiple times versus those who were never exposed at all have different patterns of the presence of certain health characteristics which include headache, stomach pain, back pain, depression, irritability or bad mood, nervousness, problems with sleep, dizziness, self-perceived health impression, injuries, days spent in physical activity per week, and the frequency of intensive physical activity, as well as risky behaviors including cigarettes and alcohol consumption. Moreover, we also hypothesized that children exposed to cyberbullying multiple times versus those who were exposed once have different patterns of the presence of certain health characteristics which include headache, stomach pain, back pain, depression, irritability or bad mood, nervousness, problems with sleep, dizziness, self-perceived health impression, injuries, days spent in physical activity per week, and the frequency of intensive physical activity, as well as risky behaviors including cigarettes and alcohol consumption.

The aim of this study is to examine cyberbullying among school-age children in Serbia and to identify risk factors associated with cyberbullying. The present study aims to extend our overall understanding of cyberbullying by specifically exploring the health characteristics and risk behavior associated with cyberbullying.

## 2. Methods

### 2.1. Study Design and Sample

This study presents a secondary analysis of the 2017 Health Behavior in School-aged Children study (HBSC) data from Serbia. The study included nationally representative sample from Serbia excluding the provinces of Kosovo and Metohija, where a total of 3267 students from 64 primary (fifth and seventh grade) and high schools (first grade) were evaluated [27]. A sampling algorithm designed by the Institute of Public Health of Serbia “Dr Milan Jovanović Batut” (IPHS) was used to select schools from four administrative regions of Serbia (Vojvodina, Belgrade, Southern and Eastern Serbia, and Šumadija and Western Serbia). The ethical approval was obtained from the IPHS Ethics Committee, (reference number 1934/1) on 3 March 2020.

The IPHS, in cooperation with the Serbian Ministry of Education, Science, and Technological Development; the Ministry of Health; as well as the World Health Organization (WHO), conducted the 2017 HBSC survey as a pilot study according to the standardized international HBSC research protocol [28]. The standardized HBSC questionnaire was translated from English to Serbian and then back to English [27]. Participation consent was obtained from the parents and schools of the children included in the HBSC study.

### 2.2. Variables of Interest in the Study

We extracted data from an electronic database on the health issues and risky behavior of a representative sample of Serbian schoolchildren (n = 3267 students) to study and compare the prevalence of health characteristics and risky behaviors between cybervictims and non-victims. In addition, we analyzed the frequency of health issues and risky behaviors among cybervictims and their relation to the number of times (once versus multiple times) cyberbullying was experienced.

Our analysis involved studying the significant differences within and between these groups and exploring health models of cybervictims. Cybervictims were identified as those who confirmed they were exposed to cyberbullying at least once in the last couple of months. The original question in the standardized HBSC questionnaire was the following [29]: “How often did they abuse you in the following way…?”

The four original answers were re-coded as below:

- “I have not been abused in this way in the past couple of months”—non-victim of cyberbullying;
- “Once or twice”—victim of cyberbullying at least once;
- “Two/three times a month; About once a week; Several times a week”;—victim of cyberbullying multiple times;
- “I don’t know/no answer”—missing.

We included the response rate for each question in the table instead of presenting missing data.

We used regression modeling to compare:

- Non-victims to those who were cyberbullied at least once (Model 1);
- Non-victims to those who were cyberbullied multiple times (Model 2);
- Those who were cyberbullied at least once to those who were cyberbullied multiple times (Model 3).

We examined health issues and risky behavior based on the following data:

The self-assessed overall health of the schoolchildren was described using their responses on a 4-point Likert scale (1 = bad, 2 = good, 3 = very good, and 4 = excellent).

#### Health Characteristics

We examined a range of health issues such as headaches, stomach pain, back pain, depression, irritability, nervousness, sleep problems, and dizziness over the past six months by identifying their frequency on a 3-point Likert scale (1 = rarely/never, 2 = almost every week, and 3 = almost every day).

The severity of injuries was examined by exploring answers regarding the need for medical assistance. The original question was: “During the last 12 months how many times have you been injured and needed physician or nurse attention?”, and responses were categorized as follows: Over the last 12 months, I had (on a 5-point Likert scale): 1 = no injuries; 2 = once; 3 = twice; 4 = three times; and 5 = four and more times.

The weight of school-age children was analyzed using body mass index (BMI) calculated from their height and weight data. BMI categories include underweight, normal weight, overweight, and obese.

Given the importance of physical activity for mental and physical perception as well as quality of life [20,30,31], we were interested in its intensity, which was addressed in the survey with two questions:

- “During the past week, how many days have you spent in physical activity at least one hour daily?”, and possible responses were ranked on a 4-point Likert scale: 1 = none; 2 = 1–2 days; 3 = 3–4 days; and 4 = 5–7 days.
- “During your free time, how frequently have you exercised intensely with a breath loss or getting sweaty?”, and responses were ranked on a 4-point Likert scale: 1 = rarely/never; 2 = once monthly; 3 = once weekly; and 4 = several times per week/every day.

To assess the smoking status, we asked [28]: “How often (if so, in days) did you smoke cigarettes?”

The subcategories included [28]:

- “Ever in life?”, and answers were given on a 5-point Likert scale from 1 to 5, where 1 = never; 2 = 1–2 days; 3 = 3–5 days; 4 = 6–9 days; and 5 = 10 or more days.
- “During the last 30 days?”, and answers were ranked on a 5-point Likert scale: 1 = I do not smoke; 2 = 1–2 days; 3 = 3–5 days; 4 = 6–9 days; and 5 = 10 or more days.

Further exploration was conducted into the smoking habits of smokers with the following question: “How often do you currently smoke cigarettes?” The answer options were ranked on a 3-point Likert scale: 1 = “less than once weekly”, 2 = “at least once weekly but not everyday”, and 3 = “everyday”.

The next risk behavior we were interested in was alcohol abuse. The participants were asked about their history of alcohol abuse, including whether they had ever consumed excessive amounts of alcohol. The answers to the original question were “Did you drink so much alcohol so that you got really drunk?” [28].

- “Ever in life?” Answers were ranked on a 5-point Likert scale: 1 = no, never; 2 = yes, once; 3 = yes, 2/3 times; 4 = yes, 4–10 times; and 5 = yes, >10 times.
- “During the last 30 days?” Answers were ranked on a 5-point Likert scale: 1 = no, never; 2 = yes, once; 3 = yes, 2/3 times; 4 = yes, 4–10 times; and 5 = yes, >10 times.

An additional question helped us determine the amount of alcohol consumed: “When you drink alcohol, in a typical day how many alcoholic beverages do you drink?”, and offered answers were ranked on a 3-point Likert scale: 1 = ≤1 drink; 2 = 2/3 drinks; and 3 = ≥4 drinks [28].

### 2.3. Statistical Analysis

In addition to the absolute and relative numbers (n and %), the prevalence of variables was presented as percentages and 95% confidence interval (CI).

Pearson chi-square test was used to assess the presence of statistically significant differences between and within the groups of non-victims and cybervictims. Moreover, three statistical models of the correlation between the explanatory variables and outcome variables were analyzed using the univariate and multivariate regression modelling and described by cross-odds ratio (OR) with a 95% CI [28]:

- In Model 1, the reference was “never exposed (none) (0)” versus “exposed at least once (1)”.
- In Model 2, the reference was “never exposed (none) (0)” versus “exposed multiple times (1)”.
- In Model 3, the reference was “exposed at least once (0)” versus “exposed multiple times (1)”.

All statistical analyses were performed by the SPSS software package, version 22.0 (2018). Statistical significance was considered as values of *p* < 0.05.

## 3. Results

### 3.1. Cybervictims versus Non-Victims: Health Issues and Risky Behavior

Although health characteristics and risky behaviors significantly differed in the studied population of 3267 school-age children, this was not always the case among the victims of cyberbullying. Most school-age children in Serbia have a normal BMI; are physically active; do not smoke or drink excessively; rarely or never experience headaches, stomach pain, back pain, depression, problems with sleep, dizziness, and injuries; and describe their health as excellent (Table 1). However, they felt nervousness, irritability, or bad moods almost every day. In Supplementary Materials S1 we present cyberbullying exposure by age, sex and school grade in Serbia.

According to Table 1 and Table 2, the prevalence of cyberbullying victims did not vary significantly across different BMIs, exercise intensities, smoking habits, smoking frequencies among smokers, or amounts of alcohol consumed among drinkers. Still, the highest prevalence of victimization was among overweight students, with more than one in seven being affected.

The prevalence of cybervictims among school-age children who experience daily headaches, stomach pain, and back pain was just under one in five (Table 1). Furthermore, schoolchildren who reported depression and dizziness had a cybervictimization prevalence rate of about one in five. Participants who experienced daily nervousness and problems with sleep had a cybervictimization prevalence rate of just below one in six, and those who experienced irritability and bad moods almost every day had a cybervictimization prevalence rate of about one in six.

One in five children, regardless of their perceived health, had experienced cybervictimization. However, this number increased to one in four for those who had suffered from four or more injuries requiring medical attention over the course of 12 months. Participants that were not active for at least one hour a day during the last seven days were the most frequently exposed to cyberbullying with a prevalence of just below one in five, while those who exercised intensely once in a month were frequently exposed to cyberbullying with a prevalence of around one in six individuals (Table 1).

About one in three children exposed to cyberbullying had smoked cigarettes three to five days in last 30 days (Table 2). Considering daily alcohol consumption habits, the most prevalent cybervictims were those who consumed four or more drinks, at a rate of just above one in five (Table 2).

### 3.2. Cybervictimization Once versus Multiple Times: Health Issues and Risky Behavior

The prevalence of cybervictimization at least once versus multiple times was higher among overweight schoolchildren; those who had almost daily headaches, stomach and back pain, depression, irritability or bad mood, nervousness, sleep problems, dizziness and were not physically active; those who did get really drunk, and among smokers (Table 1 and Table 2). On the contrary, the prevalence of cybervictimization at least once versus multiple times was lower among those who described their health as bad, who were injured multiple times in the last 12 months, and who got drunk more than 10 times in the last month.

### 3.3. Health and Risky Behavior Factors Associated with Cybervictimization

Our results are showing no causal relationship between the health and risky behavior factors and cybervictimization since the health problems may coexist or exist prior to the exposure to the cyber forms of aggression.

In Model 1, headache, stomach pain, back pain, depression, irritability or bad mood, nervousness, sleep problems, and dizziness (almost every week and almost every day) were positively associated with at least one cyberbullying exposure. One or multiple injuries over the period of 12 months that required a doctor or nurse’s help, smoking cigarettes three or more days ever in life, and having gotten drunk two or more times ever in life were positively associated with at least one cyberbullying exposure. Being physically active for at least one hour a day over the course of five to seven days was negatively associated with at least one cyberbullying exposure (Table 3).

In Model 2, headache and dizziness (almost every week and almost every day) as well as stomach pain, back pain, depression, irritability or bad mood, nervousness, and sleep problems (almost every day) were positively associated with multiple exposures to cyberbullying. One or multiple injuries over the period of 12 months that required a doctor or nurse’s help; smoking cigarettes one or more days ever in life or more than five days over the period of 30 days; and getting drunk two or more times ever in life or four or more times over the period of 30 days were positively associated with at multiple exposure cyberbullying. Bad and good perceptions of health and being physically active for at least one hour a day over three to seven days were negatively associated with multiple exposures to cyberbullying (Table 3).

In Model 3, dizziness (almost every day) was positively associated with cyberbullying exposure multiple times versus at least once. Having at least three injuries over a period of 12 months that required a doctor or nurse’s help; smoking cigarettes more than five days ever in life, as well as over the period of 30 days; and getting drunk four or more times ever in life as well as over the period of 30 days were positively associated with multiple cyberbullying exposures versus at least one cyberbullying exposure. Irritability or bad mood (almost every week and almost every day) was negatively associated with cyberbullying exposure multiple times versus at least once (Table 3). In Supplementary Materials S2, we present the univariate logistic regression coefficient (B) and *p* value.

### 3.4. Health and Risk Behavior Profiles

The health and risk behavior profile of cybervictims in Model 1 includes being 1.43 times more likely to have depression almost every day, 2.07 times more likely to have irritability or a bad mood almost every day, 2.23 times more likely to have nervousness almost every day or almost every week, 1.73 times more likely to have smoked cigarettes more than five days ever in life, 1.71 more likely to have been drunk ever in life two/three times, and 1.65 times more likely to have gotten drunk at least four times (Table 4).

The health and risk behavior profile of cybervictims in Model 2 includes being 2.27 times more likely have back pain almost every day, 2.43 times more likely to feel dizziness almost every day, 1.98 times more likely to be injured one or more times over the period of 12 months and need a doctor or nurse’s help, and 4.09 times more likely to be injured at least three times (Table 4).

The health and risk behavior profile of cybervictims in Model 3 (exposed to cyberbullying multiple times versus once) includes being 2.33 times less likely to have irritability or a bad mood almost every week and 2.38 times less likely to have it almost every day, 1.98 times more likely to have dizziness almost every day, and 2.62 times more likely to have been injured at least three times over the period of 12 months and needed a doctor or nurse’s help (Table 4). In Supplementary Materials S2, we present the multiple logistic regression coefficient B and *p* value.

## 4. Discussion

In this study, we demonstrated that significant factors associated with cyberbullying exposure in school-age children include back pain, depression, irritability or bad mood, nervousness and dizziness, as well as having been seriously injured at least once over a 12-month period requiring help from a doctor or a nurse, smoking cigarettes for at least five days overall, and getting drunk at least twice overall. More specifically, depression, irritability or bad mood, as well as nervousness are related to exposure to cyberbullying at least once.

Previous studies have already indicated that cyberbullying victims experience higher rates of depression [22,32,33] and anxiety [21,22,32,33]. Moreover, emotional problems vary in intensity and quality in adolescents who experience cyberbullying [34]. Therefore, victims of cyberbullying should be carefully assessed by healthcare professionals and closely monitored particularly for risk behavior patterns in order to timely identify these individuals and provide them with adequate treatment and counseling. This is emphasized by the fact that the negative outcomes are worse for victims of cyberbullying than for victims of traditional bullying [21].

Another health issue identified was dizziness in school-age children who were exposed to cyberbullying. Tesler et al. stated that dizziness was significantly associated with cyberbullying exposure, particularly for students in primary and junior high schools [35]. However, the possible explanation for almost daily dizziness being a strong predictor of multiple exposure to cyberbullying could be the fact that children are more frequently interacting with digital media. In line with this, Lazea et al. pointed out that dizziness was found in 14.03% of adolescents during internet use [36].

Back pain’s emergence as a factor of multiple exposures to cyberbullying could be explained by prolonged sedentary behavior, given that for children who were exposed to cyberbullying at least once, such health variable was not shown as a factor. Previous research has linked low-back pain in school-age children to physical inactivity [37]. Furthermore, a meta-analysis stressed that excessive sedentary behavior was associated with higher bullying victimization [38]. Moreover, a cross-sectional study pointed out that low-back pain was associated with moderate-to-high sedentary behaviors in girls [39]. The importance of recognizing the problem of reduced physical activity particularly in this group of children refers to the fact that the promotion of regular physical activity is beneficial, particularly in terms of psychological benefits including self-control, self-esteem, and empathy increase [40].

Injuries requiring help from a doctor or a nurse over a 12-month period were predictors of multiple exposures to cyberbullying, where three or more injuries during this period were more than four times more likely in victimized school-age children than in peers who were not cyberbullying victims.

Considering risky behavior, we have demonstrated that smoking cigarettes is a factor related to cybervictimization, particularly for victims exposed to bullying at least once with a pattern of more than five days smoking overall. Previous reports have documented that cyberbullying victimization was associated with smoking tobacco cigarettes [41,42]. Case et al. stated that predictors of tobacco use in youth are depression and anxiety [43], which was shown to be frequent in children exposed to cyberbullying. Aside from smoking cigarettes, having gotten drunk at least twice overall was shown to be a predictor particularly for being exposed to cyberbullying at least once. In the study by Rodríguez-Enríquez et al., it was found that alcohol consumption was significantly increased in adolescents who were cybervictims [44].

The prevalence of cyberbullying in Serbia is in a similar range to that in more developed countries [12]. The reason for that is probably the possibility for young people to access the Internet, and children, as well. Their usage of the Internet and mobile phones is rarely controlled by the adults, so they can reach harmful content on the Internet [12].

Lastly, let us acknowledge several study limitations. First, the findings of this study are based on self-reported data; therefore, their interpretation should consider the possibility of participants tending to provide answers that they deemed expected. Over- and underestimation of the study findings is possible due to the participants’ readiness to talk about victimization and due to the accuracy of their memory. Moreover, we did not analyze self-reported bullying experiences that are related to the real situation in children. Another limitation is contextual, that the study findings are relevant only to schoolchildren in Serbia and perhaps could be considered important to similar cultures’ social and regulative national constructs. Additionally, this study sample included participants aged between 11 and17 years; thus, further studies on schoolchildren younger than 11 years of age as well as older than 17 years should be included. The limitations adhered to in the cross-sectional study include the inability to investigate causal relations between the cyberbullying exposure and health-related outcomes. Furthermore, some recent events, such as threats, witnessing violence, or prior education about cyberbullying might have influenced their responses. It is advisable to consider that the research was conducted before the COVID-19 pandemic, whose influence on cybervictimization exposure was not studied. Owing to the isolation and changed patterns of social activities, during the COVID-19 pandemic, children developed further engagement with Internet technology, and new studies are needed to appraise its potential negative impact on the overall health of and risky behaviors in schoolchildren.

## 5. Conclusions

The study findings provide an insight into the health characteristics and risky behavior factors in cybervictims. Furthermore, the study emphasizes factors associated with repeated cyberbullying exposure. Therefore, risk factors such as back pain, depression, irritability or bad mood, nervousness and dizziness, being seriously injured at least once over a 12-month period requiring help from a doctor or a nurse, smoking cigarettes for at least five days overall, and getting drunk at least twice overall need to be targeted in preventive strategies that aim to reduce cybervictimization in the school-age population.

These findings from Serbia suggest that there is a need to investigate whether children who were aware of adverse health issues were also aware of their link to cyberbullying. In that sense, the HBSC study instrument should be improved to help children recognize the relationship between their health wellbeing and cyberbullying. The respondents’ awareness of the health implications of cyberbullying could be increased by reformulating some of the survey questions and responses. The absence of awareness could hinder schoolchildren’s ability to address both cyberbullying and its health outcomes promptly and to seek help from others and authorities. Future research should be oriented towards methods of encouraging children to talk with their parents, legal guardians, teachers, and peers about cyberbullying, victims, and perpetrators. More research is needed on the effective methods of training for school educators/teachers and parents or legal guardians to recognize signs of cyberbullying and victimization to be able to promptly take actions.

## Figures and Tables

**Table 1:** Health characteristics of school-age children, and prevalence of cybervictimization, Serbia, 2017.

Health Characteristics	Sample *n* (%)	Cybervictimization Prevalence (% and 95% Confidence Level)			*p*
		At Least Once	Multiple Times	Total	
**Body mass index—BMI** (response rate 91.5%)					
Underweight	125 (4.2)	6.4 (2.1–10.7)	4.8 (1.1–8.5)	10.8 (5.4–16.1)	0.291
Normal weight	2409 (80.6)	8.9 (7.7–10.0)	2.8 (2.2–3.5)	11.6 (10.3–12.9)	<0.001
Overweight	369 (12.3)	10.0 (7.0–13.1)	4.6 (2.5–6.7)	14.4 (10.9–18.0)	0.002
Obese	86 (2.9)	3.5 (−0.4–7.4)	4.7 (0.2–9.1)	8.0 (2.3–13.8)	0.350
*p*	<0.001	0.107	0.078	0.271	
**During the last six months, how frequently have you felt any of the following?**					
**Headache** (response rate 95.9%)					
Rarely/never	1783 (56.9)	5.8 (4.7–6.9)	2.2 (1.5–2.9)	7.9 (6.6–9.1)	<0.001
Almost every week	523 (16.7)	10.5 (7.9–13.1)	3.8 (2.2–5.5)	14.2 (11.2–17.2)	<0.001
Almost every day	828 (26.4)	13.9 (11.5–16.2)	4.7 (3.3–6.2)	18.5 (15.8–21.1)	<0.001
*p*	<0.001	<0.001	0.002	<0.001	
**Stomach pain** (response rate 95.3%)					
Rarely/never	1485 (47.7)	6.3 (5.0–7.5)	2.5 (1.7–3.3)	8.6 (7.2–10.0)	<0.001
Almost every week	1018 (32.7)	9.6 (7.8–11.4)	2.3 (1.3–3.2)	11.8 (9.8–13.8)	<0.001
Almost every day	611 (19.6)	13.6 (10.9–16.3)	6.1 (4.2–7.9)	19.5 (16.4–22.6)	<0.001
*p*	<0.001	<0.001	<0.001	<0.001	
**Back pain** (response rate 95.0%)					
Rarely/never	2027 (65.3)	6.8 (5.7–7.9)	2.1 (1.5–2.7)	8.8 (7.6–10.0)	<0.001
Almost every week	471 (15.2)	11.9 (9.0–14.8)	2.3 (1.0–3.7)	14.1 (11.0–17.3)	<0.001
Almost every day	607 (19.5)	13.0 (10.3–15.7)	6.6 (4.6–8.6)	19.5 (16.3–22.6)	<0.001
*p*	<0.001	<0.001	<0.001	<0.001	
**Depression** (response rate 93.3%)					
Rarely/never	1986 (65.2)	6.1 (5.1–7.2)	2.5 (1.8–3.1)	8.5 (7.3–9.7)	<0.001
Almost every week	410 (13.5)	11.0 (7.9–14.0)	1.7 (0.5–3.0)	12.6 (9.4–15.8)	<0.001
Almost every day	652 (21.4)	16.0 (13.1–18.8)	5.7 (3.9–7.5)	21.6 (18.4–24.7)	<0.001
*p*	<0.001	<0.001	<0.001	<0.001	
**Irritability or bad mood** (response rate 94.0%)					
Rarely/never	1058 (34.5)	3.8 (2.6–4.9)	2.5 (1.5–3.4)	6.1 (4.7–7.6)	0.040
Almost every week	753 (24.5)	7.6 (5.7–9.5)	2.0 (1.0–3.0)	9.5 (7.4–11.6)	<0.001
Almost every day	1259 (41.0)	13.7 (11.8–15.6)	4.1 (3.0–5.1)	17.7 (15.6–19.8)	<0.001
*p*	<0.001	<0.001	0.086	<0.001	
**Nervousness** (response rate 95.0%)					
Rarely/never	786 (25.3)	3.3 (2.0–4.5)	2.0 (1.0–2.9)	5.0 (3.5–6.5)	0.054
Almost every week	618 (19.9)	7.3 (5.2–9.3)	1.3 (0.4–2.2)	8.4 (6.3–10.6)	<0.001
Almost every day	1700 (54.8)	12.0 (10.5–13.5)	4.1 (3.2–5.1)	16.0 (14.2–17.7)	<0.001
*p*	<0.001	<0.001	<0.001	<0.001	
**Problems with sleep** (response rate 94.4%)					
Rarely/never	1965 (63.7)	7.0 (5.9–8.2)	2.6 (1.9–3.4)	9.5 (8.2–10.8)	<0.001
Almost every week	320 (10.4)	10.9 (7.5–14.4)	1.9 (0.4–3.4)	12.9 (9.2–16.6)	<0.001
Almost every day	799 (25.9)	12.1 (9.9–14.4)	4.1 (2.8–5.5)	16.1 (13.6–18.6)	<0.001
*p*	<0.001	<0.001	0.055	<0.001	
**Dizziness** (response rate 94.8%)					
Rarely/never	2447 (79.0)	7.8 (6.7–8.8)	2.1 (1.6–2.7)	9.8 (8.6–10.9)	<0.001
Almost every week	299 (9.7)	11.4 (7.8–15.0)	5.0 (2.5–7.5)	16.3 (12.1–20.4)	0.002
Almost every day	352 (11.4)	13.9 (10.3–17.5)	7.4 (4.7–10.1)	21.2 (17.0–25.5)	0.002
*p*	<0.001	<0.001	<0.001	<0.001	
**What do you think your health is like?** (response rate 96.2%)					
Excellent	2052 (65.3)	7.2 (6.0–8.3)	2.9 (2.2–3.7)	9.9 (8.7–11.2)	<0.001
Very good	778 (24.7)	10.2 (8.0–12.3)	2.4 (1.4–3.5)	12.5 (10.2–14.8)	<0.001
Good	268 (8.5)	15.3 (11.0–19.6)	5.6 (2.8–8.3)	20.6 (15.8–25.4)	<0.001
Bad	46 (1.5)	8.7 (0.6–16.8)	13.0 (3.3–22.8)	20.4 (9.1–31.7)	0.251
*p*	<0.001	<0.001	<0.001	<0.001	
**During the last 12 months, how many times have you been injured and needed a physician or nurse’s attention?** (response rate 99.0%)					
Over the last 12 months I had no injuries	1823 (57.5)	7.4 (6.2–8.5)	37.0 (36.4–37.4)	14.6 (13.0–16.2)	<0.001
Once	704 (22.2)	10.5 (8.2–12.8)	26.0 (24.6–27.4)	20.9 (17.9–23.9)	<0.001
Twice	332 (10.5)	10.2 (7.0–13.5)	13.0 (10.9–15.1)	20.0 (15.7–24.3)	<0.001
Three times	135 (4.3)	7.4 (3.0–11.8)	3.0 (0.5–5.5)	14.7 (8.8–20.7)	0.023
Four and more times	178 (5.6)	13.5 (8.5–18.5)	22.0 (17.2–26.8)	25.7 (20.3–33.3)	0.375
*p*	<0.001	0.008	<0.001	<0.001	
**During the past week, how many days have you engaged in physical activity at least one hour daily?** (response rate 96.5%)					
None	155 (4.9)	12.9 (7.6–18.2)	7.1 (3.1–11.1)	19.5 (13.3–25.7)	0.044
1–2 days	436 (13.8)	9.6 (6.9–12.4)	3.9 (2.1–5.7)	13.3 (10.1–16.4)	<0.001
3–4 days	776 (24.6)	9.3 (7.2–11.3)	2.2 (1.2–3.2)	11.3 (9.1–13.5)	<0.001
5–7 days	1786 (56.6)	7.7 (6.4–8.9)	2.9 (2.1–3.7)	10.5 (9.1–11.9)	<0.001
*p*	<0.001	0.035	0.003	<0.001	
**During your free time, how frequently have you exercised intensely with a loss of breath or getting sweaty?** (response rate 94.9%)					
Rarely/never	516 (16.7)	8.1 (5.8–10.5)	4.5 (2.7–6.2)	12.4 (9.6–15.3)	0.007
Once monthly	187 (6.0)	12.3 (7.6–17.0)	4.3 (1.4–7.2)	16.6 (11.2–21.9)	0.002
Once weekly	373 (12.0)	10.7 (7.6–13.9)	2.1 (0.7–3.6)	12.7 (9.4–16.1)	<0.001
Several times per week/every day	2023 (65.3)	8.2 (7.0–9.4)	2.8 (2.1–3.5)	10.9 (9.5–12.2)	<0.001
*p*	<0.001	0.056	0.051	0.093	

**Table 2:** Risky behavior of school-age children and prevalence of cybervictimization, Serbia, 2017.

Risky Behavior	Sample *n* (%)	Cybervictimization Prevalence (% and 95% Confidence Interval)			*p*
		At Least Once	Multiple Times	Total	
**How often (if so, in days) have you smoked cigarettes, ever in life?** (response rate 96.4%)					
Never	2588 (82.4)	8.0 (6.9–9.0)	2.2 (1.6–2.7)	10.1 (9.0–11.3)	<0.001
1–2 days	160 (5.1)	13.8 (8.4–19.1)	5.0 (1.6–8.4)	18.8 (12.7–24.8)	0.004
3–5 days	66 (2.1)	12.1 (4.2–20.0)	9.1 (2.2–16.0)	21.2 (11.3–31.1)	0.286
6–9 days	26 (0.8)	15.4 (1.5–29.3)	11.5 (−0.7–23.8)	26.9 (9.9–44.0)	0.342
10 and more days	299 (9.5)	11.0 (7.5–14.5)	8.0 (4.9–11.1)	19.4 (14.9–23.9)	0.084
*p*	<0.001	0.022	<0.001	<0.001	
**How often (if so, in days) did you smoke cigarettes during the last 30 days?** (response rate 95.9%)					
I don’t smoke	204 (37.9)	12.7 (8.2–17.3)	3.9 (1.3–6.6)	16.7 (11.6–21.8)	<0.001
1–2 days	60 (11.2)	15.0 (6.0–24.0)	6.7 (0.4–13.0)	21.7 (11.2–32.1)	0.071
3–5 days	32 (5.9)	21.9 (7.6–36.2)	9.4 (−0.7–19.5)	31.3 (15.2–47.3)	0.084
6–9 days	27 (5.0)	18.5 (3.9–33.2)	7.4 (−2.5–17.3)	25.9 (9.4–42.5)	0.112
10 and more days	215 (40.0)	9.3 (5.4–13.2)	9.8 (5.8–13.7)	19.1 (13.8–24.3)	0.434
*p*	<0.001	0.211	0.219	0.311	
**How often do you currently smoke cigarettes?** (response rate 98.4%)					
Less than once weekly	58 (18.6)	15.5 (6.2–24.8)	5.2 (−0.5–10.9)	20.7 (10.3–31.1)	0.034
At least once weekly but not every day	68 21.8)	13.2 (5.2–21.3)	4.4 (−0.5–9.3)	17.6 (8.6–26.7)	0.035
Every day	186 (59.6)	9.1 (5.0–13.3)	11.3 (6.7–15.8)	20.4 (14.6–26.2)	0.247
*p*	<0.001	0.339	0.130	0.873	
**Have you drank so much alcohol that you got really drunk ever in life?** (response rate 97.1%)					
No, never	2221 (70.0)	7.5 (6.4–8.6)	1.9 (1.3–2.5)	9.4 (8.2–10.6)	<0.001
Yes, once	474 (14.9)	13.1 (10.0–16.1)	4.2 (2.4–6.0)	17.3 (13.9–20.7)	<0.001
Yes, 2/3 times	232 (7.3)	13.4 (9.0–17.7)	3.0 (0.8–5.2)	16.4 (11.6–21.1)	<0.001
Yes, 4–10 times	105 (3.3)	5.7 (1.3–10.2)	6.7 (1.9–11.4)	12.4 (6.1–18.7)	0.387
Yes, >10 times	141 (4.4)	7.1 (2.9–11.3)	17.0 (10.8–23.2)	24.1 (17.1–31.2)	0.005
*p*	<0.001	<0.001	<0.001	<0.001	
**Did you drink so much alcohol so that you got really drunk during the last 30 days?** (response rate 96.5%)					
No, never	533 (58.0)	11.6 (8.9–14.4)	3.8 (2.1–5.4)	15.4 (12.3–18.4)	<0.001
Yes, once	227 (24.7)	15.9 (11.0–20.8)	3.5 (1.1–5.9)	19.4 (14.2–24.5)	<0.001
Yes, 2/3 times	81 (8.8)	6.2 (0.9–11.4)	7.4 (1.7–13.1)	13.6 (6.1–21.0)	0.377
Yes, 4–10 times	24 (2.6)	12.5 (−0.7–25.7)	20.8 (4.6–37.1)	33.3 (14.5–52.2)	0.219
Yes, >10 times	54 (5.9)	3.7 (−1.3–8.7)	29.6 (17.5–41.8)	33.3 (20.8–45.9)	<0.001
*p*	<0.001	0.049	<0.001	0.003	
**When you drink alcohol, in a typical day, how many alcoholic beverages do you drink?** (response rate 74.6%)					
≤1 drink	117 (16.5)	13.7 (7.4–19.9)	3.4 (0.1–6.7)	17.1 (10.3–23.9)	0.003
2/3 drinks	304 (43.0)	9.9 (6.5–13.2)	4.3 (2.0–6.6)	14.1 (10.2–18.1)	0.004
≥4 drinks	286 (40.5)	12.2 (8.4–16.0)	9.4 (6.1–12.8)	21.7 (16.9–26.5)	0.141
*p*	<0.001	0.473	0.013	0.056	

**Table 3:** Health characteristics and risky behavior associated with cybervictimization, school-age children in Serbia, 2017.

Variables	Univariate Logistic Regression (Odds Ratio—OR and Confidence Interval—CI)		
	Model 1: Non-Cybervictim versus Cybervictim at Least Once	Model 2: Non-Cybervictim versus Cybervictim Multiple Times	Model 3: Cybervictim at Least Once versus Multiple Times
	OR (95% CI)	OR (95% CI)	OR (95% CI)
**Health characteristics**			
**Body mass index**			
Normal weight	/	1	1
Underweight	/	1.69 (0.72–3.98)	2.36 (0.79–7.04)
Overweight	/	1.69 (0.98–2.91)	1.45 (0.77–2.73)
Obese	/	1.58 (0.56–4.45)	4.20 (0.92–19.2)
**During last six months, how frequently have you felt any of the following?**			
**Headache**			
Rarely/never	1	1	1
Almost every week	1.96 *** (1.39–2.76)	1.88 * (1.09–3.25)	0.96 (0.51–1.80)
Almost every day	2.72 *** (2.05–3.60)	2.44 *** (1.55–3.83)	0.90 (0.53–1.50)
**Stomach pain**			
Rarely/never	1	1	1
Almost every week	1.59 ** (1.18–2.14)	0.94 (0.55–1.59)	0.59 (0.33–1.07)
Almost every day	2.46 *** (1.80–3.37)	2.76 *** (1.73–4.40)	1.12 (0.65–1.93)
**Back pain**			
Rarely/never	1	1	1
Almost every week	1.85 *** (1.34–2.58)	1.17 (0.60–2.29)	0.63 (0.30–1.31)
Almost every day	2.17 *** (1.61–2.91)	3.52 *** (2.26–5.47)	1.63 (0.97–2.71)
**Depression**			
Rarely/never	1	1	1
Almost every week	1.87 ** (1.30–2.68)	0.72 (0.33–1.61)	1.13 (0.68–1.86)
Almost every day	3.03 *** (2.29–4.00)	2.68 *** (1.73–4.16)	0.44 (0.18–1.05)
**Irritability or bad mood**			
Rarely/never	1	1	1
Almost every week	2.08 ** (1.37–3.15)	0.84 (0.44–1.60)	0.45 ** (0.25–0.81)
Almost every day	4.15 *** (2.91–5.91)	1.88 * (1.16–3.04)	0.40 * (0.19–0.86)
**Nervousness**			
Rarely/never	1	1	1
Almost every week	2.38 ** (1.44–3.92)	0.70 (0.30–1.67)	1.75 (0.87–3.51)
Almost every day	4.27 *** (2.79–6.53)	2.44 *** (1.39–4.29)	0.52 (0.23–1.15)
**Problems with sleep**			
Rarely/never	1	1	1
Almost every week	1.61 * (1.09–2.39)	0.73 (0.31–1.73)	0.46 (0.18–1.15)
Almost every day	1.87 *** (1.42–2.45)	1.68 * (1.08–2.63)	0.90 (0.54–1.50)
**Dizziness**			
Rarely/never	1	1	1
Almost every week	1.58 * (1.07–2.33)	2.54 ** (1.41–4.59)	1.61 (0,82–3.18)
Almost every day	2.05 *** (1.46–2.88)	3.98 *** (2.45–6.48)	1.94 * (1.10–3.41)
**What do you think your health is like?**			
Excellent	1	1	1
Very good	1.74 (0.59–5.16)	0.42 (0.16–1.17)	3.68 (1.00–13.5)
Good	1.05 (0.36–3.02)	0.20 *** (0.08–0.48)	0.90 (0.46–1.74)
Bad	0.72 (0.25–2.04)	0.17 *** (0.06–0.45)	0.59 (0.33–1.06)
**During the last 12 months, how many times have you been injured and needed a physician or nurse’s attention?**			
Over the last 12 months I had no injuries	1	1	1
Once or twice	1.50 ** (1.15–1.96)	1.96 ** (1.24–3.09)	1.31 (0.78–2.19)
Three or more times	1.65 * (1.11–2.46)	4.40 *** (2.60–7.42)	2.66 **(1.42–5.01)
**During the past week, how many days have you engaged in physical activity at least one hour daily?**			
None	1	1	1
1–2 days	0.69 (0.39–1.22)	0.51 (0.23–1.12)	0.74 (0.29–1.86)
3–4 days	0.65 (0.38–1.11)	0.37 ** (0.19–0.72)	0.43 (0.17–1.06)
5–7 days	0.53 * (0.32–0.88)	0.28 ** (0.13–0.61)	0.69 (0.31–1.54)
**During your free time, how frequently have you exercised intensely with a loss of breath or getting sweaty?**			
Rarely/never	1	1	1
Once monthly	1.58 (0.92–2.72)	1.01 (0.44–2.29)	1.62 (0.90–2.93)
Once weekly	1.32 (0.84–2.09)	0.48 (0.21–1.09)	1.03 (0.44–2.44)
Several times per week/every day	0.99 (0.70–1.41)	0.61 (0.37–1.00)	0.59 (0.26–1.34)
**Risky behavior**			
**How often (if so, in days) have you smoked cigarettes ever in life?**			
Never	1	1	1
1–2 days	1.74 (0.81–3.73)	2.56 * (1.19–5.47)	1.34 (0.57–3.17)
3–5 days	1.59 * (1.10–2.30)	4.15 ** (2.58–6.69)	2.76 (0.92–8.28)
≥6 days	1.91 ** (1.19–3.07)	4.79 ** (1.98–11.6)	2.61 ** (1.41–4.65)
**How often (if so, in days) did you smoke cigarettes during the last 30 days?**			
I don’t smoke	1	1	1
1–2 days	1.25 (0.55–2.85)	1.81 (0.52–6.27)	1.44 (0.35–5.97)
3–5 days	2.08 (0.81–5.36)	2.90 (0.72–11.7)	1.39 (0.29–6.68)
≥6 days	0.84 (0.47–1.51)	2.52 * (1.10–5.78)	2.99 * (1.13–7.92)
**How often do you currently smoke cigarettes?**			
Less than once weekly	1	1	1
At least once weekly but not every day	0.82 (0.30–2.24)	0.82 (0.16–4.28)	1.00 (0.16–6.35)
Every day	0.59 (0.25–1.41)	2.18 (0.62–7.63)	3.71 (0.87–15.9)
**Have you drank so much alcohol that you got really drunk ever in life?**			
No, never	1	1	1
Yes, once	1.03 (0.60–1.75)	1.69 (0.75–3.80)	1.25 (0.68–2.28)
Yes, 2/3 times	1.97 * (1.11–3.50)	2.39 ** (1.39–4.10)	0.87 (0.36–2.11)
Yes, ≥4 times	1.99 * (1.05–3.75)	7.29 *** (4.49–11.8)	7.48 *** (3.75–14.9)
**Did you drink so much alcohol so that you got really drunk during the last 30 days?**			
No, never	1	1	1
Yes, once	1.43 (0.92–2.23)	0.99 (0.43–2.28)	0.69 (0.28–1.72)
Yes, 2/3 times	0.50 (0.20–1.34)	1.93 (0.75–4.98)	3.72 (1.03–13.5)
Yes, ≥4 times	0.70 (0.27–1.82)	9.11 *** (4.63–17.9)	13.02 *** (4.34–39.0)
**When you drink alcohol, in a typical day, how many alcoholic beverages do you drink?**			
≤1 drink	1	1	1
2/3 drinks	0.70 (0.36–1.34)	1.21 (0.39–3.79)	1.73 (0.49–6.20)
≥4 drinks	0.95 (0.50–1.79)	2.92 (1.00–8.58)	3.09 (0.93–10.3)

OR—odds ratio; CI—confidence interval; * *p* < 0.05; ** *p* < 0.01; *** *p* < 0.001.

**Table 4:** Health and risky behavior profiles of cybervictims, school-age children in Serbia, 2017.

Variables	Multivariate Logistic Regression (odds ratio—OR and confidence interval—CI)		
	Model 1: Non-Cybervictim versus Cybervictim at Least Once	Model 2: Non-Cybervictim versus Cybervictim Multiple Times	Model 3: Cybervictim at Least Once versus Multiple Times
**Health characteristics**			
**Body mass index**			
Normal weight	/	/	/
Underweight	/	/	/
Overweight	/	/	/
Obese	/	/	/
**During the last six months, how frequently have you felt any of the following?**			
**Headache**			
Rarely/never	1	1	/
Almost every week	1.27 (0.88–1.83)	1.84 (0.98–3.45)	/
Almost every day	1.40 (0.99–1.98)	0.92 (0.48–1.76)	/
**Stomach pain**			
Rarely/never	1	1	/
Almost every week	1.17 (0.85–1.81)	0.74 (0.40–1.34)	/
Almost every day	1.32 (0.92–1.91)	1.24 (0.68–2.26)	/
**Back pain**			
Rarely/never	1	1	/
Almost every week	1.39 (0.98–1.98)	0.93 (0.45–1.43)	/
Almost every day	1.31 (0.94–1.82)	2.27 ** (1.32–3.91)	/
**Depression**			
Rarely/never	1	1	/
Almost every week	1.21 (0.82–1.78)	0.51 (0.19–1.35)	/
Almost every day	1.43 * (1.02–2.01)	1.50 (0.82–2.76)	/
**Irritability or bad mood**			
Rarely/never	1	1	1
Almost every week	1.55 (0.99–2.42)	0.74 (0.35–1.58)	0.43 ** (0.23–0.80)
Almost every day	2.07 ** (1.33–3.23)	0.80 (0.40–1.63)	0.42 ** (0.19–0.92)
**Nervousness**			
Rarely/never	1	1	/
Almost every week	2.23 ** (1.28–3.90)	0.66 (0.24–1.78)	/
Almost every day	2.23 ** (1.32–3.80)	1.51 (0.72–3.16)	/
**Problems with sleep**			
Rarely/never	1	1	/
Almost every week	1.20 (0.79–1.82)	0.60 (0.23–1.56)	/
Almost every day	1.04 (0.76–1.42)	0.80 (0.46–1.38)	/
**Dizziness**			
Rarely/never	1	1	1
Almost every week	1.02 (0.67–1.56)	1.89 (0.97–3.68)	1.70 (0.84–3.44)
Almost every day	0.97 (0.65–1.45)	2.43 ** (1.25–4.73)	1.98 * (1.06–3.68)
**What do you think your health is like?**			
Excellent	/	1	/
Very good	/	0.62 (0.20–1.93)	/
Good	/	0.39 (0.13–1.20)	/
Bad	/	0.59 (0.20–1.70)	/
**During the last 12 months, how many times have you been injured and needed a physician or nurse’s attention?**			
Over the last 12 months I had no injuries	1	1	1
Once or twice	1.31 (0.98–1.74)	1.98 * (1.16–3.39)	1.38 (0.79–2.42)
Three and more times	0.14 (0.73–1.79)	4.09 *** (2.18–7.66)	2.62 ** (1.32–5.18)
**During the past week, how many days have you engaged in physical activity at least one hour daily?**			
None	1	1	/
1–2 days	0.83 (0.46–1.49)	0.80 (0.33–1.98)	/
3–4 days	0.84 (0.49–1.46)	0.57 (0.24–1.38)	/
5–7 days	0.83 (0.49–1.40)	0.61 (0.27–1.37)	/
**During your free time, how frequently have you exercised intensely with a loss of breath or getting sweaty?**			
Rarely/never	/	/	/
Once monthly	/	/	/
Once weekly	/	/	/
Several times per week/every day	/	/	/
**Risky behavior**			
**How often (if so, in days) have you smoked cigarettes ever in life?**			
Never	1	1	1
1–2 days	1.44 (0.66–3.14)	0	0
3–5 days	1.37 (0.89–2.10)	0	0
≥6 days	1.73 * (1.06–2.82)	0	0
**How often (if so, in days) did you smoke cigarettes during the last 30 days?**			
I don’t smoke	/	1	1
1–2 days	/	1.30 (0.25–6.70)	1.67 (0.23–12.2)
3–5 days	/	5.47 (0.93–32.3)	5.04 (0.45–56.1)
≥6 days	/	4.08 (0.88–18.9)	6.96 (0.86–56.4)
**How often do you currently smoke cigarettes?**			
Less than once weekly	/	/	/
At least once weekly but not every day	/	/	/
Every day	/	/	/
**Have you drank so much alcohol that you got really drunk ever in life?**			
No, never	1	1	1
Yes, once	0.81 (0.46–1.44)	0.29 (0.05–1.63)	0
Yes, 2/3 times	1.71 ** (1.23–2.38)	1.88 (0.34–4.04)	0.31 (0.04–2.32)
Yes, ≥4 times	1.65 * (1.05–2.59)	1.98 (0.98–4.04)	3.03 (0.50–18.3)
**Did you drink so much alcohol so that you got really drunk during the last 30 days?**			
No, never	/	1	1
Yes, once	/	0.29 (0.06–1.40)	0.31 (0.04–2.27)
Yes, 2/3 times	/	1.55 (0.38–6.37)	1.52 (0.19–12.4)
Yes, ≥4 times	/	4.90 (1.29–18.5)	4.49 (0.54–37.1)
**When you drink alcohol, in a typical day, how many alcoholic beverages do you drink?**			
≤1 drink	/	/	/
2/3 drinks	/	/	/
≥4 drinks	/	/	/

* *p* < 0.05; ** *p* < 0.01; *** *p* < 0.001. (0—no confidence interval; 1—reference value; /—not significant coefficient in univariate logistic regression).

## Data Availability

The data presented in this study are available on request from the corresponding author.

## Supplementary Materials

The following supporting information can be downloaded at: https://www.mdpi.com/article/10.3390/children11020172/s1. Supplementary Material S1: Cyberbullying exposure (n, %) by age, sex and school grade, Serbia, 2017. Supplementary Material S2: Logistic regression analysis, coefficients B and p in each model of cyberbulling exposure (Model 1); Logistic regression analysis, coefficients B and p in each model of cyberbulling exposure (Model 2); Logistic regression analysis, coefficients B and p in each model of cyberbulling exposure (Model 3).
