# Supplementary material for: Recognizing the Frequency of Exposure to Cyberbullying in Children: The Results of the National HBSC Study in Serbia
Source: Children (Basel). 2024 Jan 29;11(2):172. doi: 10.3390/children11020172 (PMC10887102; doi:10.3390/children11020172)
Supplement: Supplementary file 1 [file children-11-00172-s001.zip › children-2775903-supplementary.pdf]

Supplementary Material S1. Cyberbullying exposure (n, %) by age, sex and school grade, Serbia, 2017

Characteristics	Cyberbullying exposure, n (%)			
	Never exposed	Once to two times	Multiple times	Total
**Sex**				
Male	1457 (51.9)	112 (40.4)	64 (62.7)	1633 (51.3)
Female	1350 (48.1)	165 (59.8)	38 (37.3)	1553 (48.7)
**Age (years)**				
11 – 12	787 (28.0)	45 (16.2)	22 (21.6)	854 (26.8)
13 – 14	805 (28.7)	96 (34.7)	30 (29.4)	931 (29.2)
15 – 17	1215 (28.7)	136 (49.1)	50 (49.0)	1401 (44.0)
**School grade**				
Fifth grade primary school	805 (28.7)	46 (16.6)	25 (24.5)	876 (27.5)
Seventh grade primary school	797 (28.4)	98 (35.4)	27 (26.5)	922 (28.9)
First grade high school	291 (10.4)	36 (13.0)	15 (14.7)	342 (10.7)
First grade vocational school	914 (32.6)	97 (35.0)	35 (34.3)	1046 (32.8)

Supplementary Material S2

Logistic regression analysis, coefficients B and p in each model of cyberbulling exposure (Model 1)

Variables (Model 1)	Univariate logistic regression		Multivariate logistic regression	
	B	p	B	p
**Health characteristics**				
**Body mass index**				
Normal weight	1			
Underweight	-0.33	0.371		
Overweight	0.16	0.410		
Obese	-0.97	0.100		
**During last six months how frequent you felt any of the following?**				
**Headache**				
Rarely/never	1		1	
Almost every week	0.67	<0.001	0.24	0.201
Almost every day	1.00	<0.001	0.34	0.055
**Stomach pain**				
Rarely/never	1		1	
Almost every week	0.45	0.002	0.16	0.329
Almost every day	0.90	<0.001	0.28	0.137
**Back pain**				
Rarely/never	1		1	
Almost every week	0.62	<0.001	0.33	0.064
Almost every day	0.77	<0.001	0.27	0.112
**Depression**				

Rarely/never	1		1	
Almost every week	0.63	0.001	0.19	0.344
Almost every day	1.11	<0.001	0.36	0.040
**Irritability or bad mood**				
Rarely/never	1		1	
Almost every week	0.73	0.001	0.44	0.056
Almost every day	1.42	<0.001	0.73	0.001
**Nervousness**				
Rarely/never	1		1	
Almost every week	0.87	0.001	0.80	0.005
Almost every day	1.45	<0.001	0.80	0.003
**Problems with a sleep**				
Rarely/never	1		1	
Almost every week	0.48	0.017	0.18	0.394
Almost every day	0.62	<0.001	0.04	0.790
**Dizziness**				
Rarely/never	1		1	
Almost every week	0.46	0.021	0.02	0.977
Almost every day	0.72	<0.001	-0.03	0.894
**What do you think your health is like?**				
Excellent	1			
Very good	0.55	0.317		
Good	0.05	0.934		
Bad	-0.33	0.533		
**During the last 12 months how many times have you been injured and needed physician or nurse attention?**				
Over the last 12 months I had no injuries	1		1	
Once or Twice	0.40	0.003	0.27	0.068
Three and more times	0.50	0.014	0.14	0.553
**During the past week, how many days have you spent in physical activity at least one our daily?**				
None	1		1	
1-2 days	-0.37	0.203	-0.19	0.528
3-4 days	-0.43	0.112	-0.17	0.540
5-7 days	-0.63	0.014	-0.19	0.478
**During your free time, how frequent have you exercise intensely with a breath loss or getting sweaty?**				
Rarely/never	1			
Once monthly	0.46	0.095		

Once weekly	0.28	0.231		
Several times per week/ Every day	-0.01	0.954		
	**Risky behavior**			
**How often (if so, in days) did you smoke cigarettes, ever in life?**				
Never	1		1	
1-2 days	0.55	0.153	0.37	0.354
3-5 days	0.46	0.014	0.31	0.149
≥6 days	0.65	0.007	0.55	0.028
**How often (if so, in days) did you smoke cigarettes, during the last 30days?**				
I don’t smoke	1			
1-2 days	0.23	0.593		
3-5 days	0.73	0.129		
≥6 days	-0.17	0.567		
**How often do you currently smoke cigarettes?**				
Less than Once weekly	1			
At least Once weekly but not everyday	-0.20	0.701		
Everyday	-0.53	0.232		
**Did you drink so much alcohol so that you got really drunk, ever in life?**				
No, never	1		1	
Yes, once	0.03	0.924	-0.21	0.476
Yes, 2/3 times	0.68	0.021	0.54	0.001
Yes, ≥4 times	0.69	0.034	0.50	0.029
**Did you drink so much alcohol so that you got really drunk, during the last 30 days?**				
No, never	1			
Yes, once	0.36	0.112		
Yes, 2/3 times	-0.66	0.175		
Yes, ≥4 times	-0.36	0.463		
**When you drink alcohol, in a typical day how many alcoholic beverages do you drink?**				
≤1 drink	1			
2/3 drinks	-0.36	0.276		
≥4 drinks	-0.05	0.868		

Logistic regression analysis, coefficients B and p in each model of cyberbulling exposure (Model 2)

**Variables (Model 2)**	**Univariate logistic regression**		**Multivariate logistic regression**	
	**B**	**P**	**B**	**P**
**Health characteristics**				
**Body mass index**				
Normal weight	1			
Underweight	0.53	0.229		
Overweight	0.52	0.059		
Obese	0.46	0.383		
**During last six months how frequent you felt any of the following?**				
**Headache**				
Rarely/never	1		1	
Almost every week	0.63	0.024	0.61	0.057
Almost every day	0.89	<0.001	-0.08	0.803
**Stomach pain**				
Rarely/never	1		1	
Almost every week	-0.06	0.815	-0.31	0.318
Almost every day	1.02	<0.001	0.22	0.485
**Back pain**				
Rarely/never	1		1	
Almost every week	0.16	0.648	-0.07	0.853
Almost every day	1.26	<0.001	0.82	0.003
**Depression**				
Rarely/never	1		1	
Almost every week	-0.32	0.429	-0.68	0.175
Almost every day	0.99	<0.001	0.41	0.189
**Irritability or bad mood**				
Rarely/never	1		1	
Almost every week	-0.17	0.596	-0.30	0.440
Almost every day	0.63	0.010	-0.22	0.546
**Nervousness**				
Rarely/never	1		1	
Almost every week	-0.35	0.427	-0.42	0.406
Almost every day	0.89	<0.001	0.41	0.279
**Problems with a sleep**				
Rarely/never	1		1	
Almost every week	-0.31	0.478	-0.51	0.294
Almost every day	0.52	0.022	-0.23	0.421
**Dizziness**				
Rarely/never	1		1	
Almost every week	0.93	0.002	0.63	0.064
Almost every day	1.38	<0.001	0.89	0.009

**What do you think your health is like?**				
Excellent	1		1	
Very good	-0.86	0.097	-0.48	0.405
Good	-1.63	<0.001	-0.94	0.101
Bad	-1.79	<0.001	-0.53	0.326
**During the last 12 months how many times have you been injured and needed physician or nurse attention?**				
Over the last 12 months I had no injuries	1		1	
Once or Twice	0.67	0.004	0.68	0.013
Three and more times	1.48	<0.001	1.41	<0.001
**During the past week, how many days have you spent in physical activity at least one our daily?**				
None	1		1	
1-2 days	-0.68	0.091	-0.22	0.634
3-4 days	-1.00	0.004	-0.56	0.211
5-7 days	-1.38	0.001	-0.496	0.233
**During your free time, how frequent have you exercise intensely with a breath loss or getting sweaty?**				
Rarely/never	1			
Once monthly	0.01	0.989		
Once weekly	-0.73	0.081		
Several times per week/ Every day	-0.50	0.051		
		**Risky behavior**		
**How often (if so, in days) did you smoke cigarettes, ever in life?**				
Never	1		1	
1-2 days	0.94	0.016	17.3	0.999
3-5 days	1.42	0.001	17.6	0.999
≥6 days	1.57	0.001	16.1	0.999
**How often (if so, in days) did you smoke cigarettes, during the last 30 days?**				
I don’t smoke	1		1	
1-2 days	0.59	0.350	0.26	0.754
3-5 days	1.06	0.136	1.70	0.061
≥6 days	0.92	0.029	1.41	0.073

**How often do you currently smoke cigarettes?**				
Less than Once weekly	1			
At least Once weekly but not everyday	-0.20	0.815		
Everyday	0.78	0.224		
**Did you drink so much alcohol so that you got really drunk, ever in life?**				
No, never	1		1	
Yes, once	0.52	0.206	-1.24	0.160
Yes, 2/3 times	0.87	0.002	0.16	0.794
Yes, ≥4 times	1.99	<0.001	0.69	0.058
**Did you drink so much alcohol so that you got really drunk, during the last 30 days?**				
No, never	1		1	
Yes, once	-0.01	0.973	-1.24	0.124
Yes, 2/3 times	0.66	0.172	0.44	0.547
Yes, ≥4 times	2.21	<0.001	1.59	0.019
**When you drink alcohol, in a typical day how many alcoholic beverages do you drink?**				
≤1 drink	1			
2/3 drinks	0.19	0.746		
≥4 drinks	1.07	0.051		

Logistic regression analysis, coefficients B and p in each model of cyberbulling exposure (Model 3)

**Variables (Model 3)**	**Univariate logistic regression**		**Multivariate logistic regression**	
	**B**	**p**	**B**	**p**
**Health characteristics**				
**Body mass index**				
Normal weight	1			
Underweight	0.86	0.124		
Overweight	0.37	0.256		
Obese	1.43	0.065		
**During last six months how frequent you felt any of the following?**				
**Headache**				
Rarely/never	1			
Almost every week	-0.04	0.900		
Almost every day	-0.11	0.676		

**Stomach pain**				
Rarely/never	1			
Almost every week	-0.53	0.081		
Almost every day	0.11	0.682		
**Back pain**				
Rarely/never	1			
Almost every week	-0.46	0.216		
Almost every day	0.49	0.063		
**Depression**				
Rarely/never	1			
Almost every week	0.12	0.635		
Almost every day	-0.83	0.065		
**Irritability or bad mood**				
Rarely/never	1		1	
Almost every week	-0.79	0.008	-0.84	0.008
Almost every day	-0.90	0.019	-0.87	0.003
**Nervousness**				
Rarely/never	1			
Almost every week	0.56	0.115		
Almost every day	-0.66	0.107		
**Problems with a sleep**				
Rarely/never	1			
Almost every week	-0.79	0.094		
Almost every day	-0.10	0.693		
**Dizziness**				
Rarely/never	1		1	
Almost every week	0.48	0.169	0.53	0.139
Almost every day	0.66	0.022	0.68	0.032
**What do you think your health is like?**				
Excellent	1			
Very good	1.30	0.050		
Good	-0.11	0.746		
Bad	-0.53	0.076		
**During the last 12 months how many times have you been injured and needed physician or nurse attention?**				
Over the last 12 months I had no injuries	1		1	
Once or Twice	0.27	0.308	0.32	0.257
Three and more times	0.98	0.002	0.96	0.006
**During the past week, how many days have you spent in physical activity at least one our daily?**				

None	1			
1-2 days	-0.31	0.517		
3-4 days	-0.85	0.067		
5-7 days	-0.37	0.365		
**During your free time, how frequent have you exercise intensely with a breath loss or getting sweaty?**				
Rarely/never	1			
Once monthly	0.48	0.109		
Once weekly	0.03	0.944		
Several times per week/ Every day	-0.52	0.210		
	**Risky behavior**			
**How often (if so, in days) did you smoke cigarettes, ever in life?**				
Never	1		1	
1-2 days	0.29	0.508	18.5	0.999
3-5 five days	1.02	0.070	19.7	0.999
≥6 days	0.96	0.001	17.4	0.999
**How often (if so, in days) did you smoke cigarettes, during the last 30days?**				
I don't smoke	1		1	
1-2 days	0.37	0.612	0.50	0.614
3-5 days	0.33	0.679	1.62	0.188
≥6 days	1.10	0.028	1.94	0.069
**How often do you currently smoke cigarettes?**				
Less than Once weekly	1			
At least Once weekly but not everyday	0	0.999		
Everyday	1.37	0.078		
**Did you drink so much alcohol so that you got really drunk, ever in life?**				
No, never	1		1	
Yes, once	0.22	0.478	17.4	0.999
Yes, 2/3 times	-0.14	0.761	-1.18	0.253
Yes, >4 times	2.01	<0.001	1.11	0.227
**Did you drink so much alcohol so that you got really drunk, during the last 30 days?**				
No, never	1		1	
Yes, once	-0.37	0.426	-1.19	0.246

Yes, 2/3 times	1.31	0.046	0.42	0.695
Yes, >4 times	2.57	<0.001	1.50	0.164
**When you drink alcohol, in a typical day how many alcoholic beverages do you drink?**				
≤1 drink	1			
2/3 drinks	0.55	0.398		
>4 drinks	1.13	0.067		
